# Therapeutic efficacy of different brands of albendazole against soil transmitted helminths among students of Mendera Elementary School, Jimma, Southwest Ethiopia

**DOI:** 10.11604/pamj.2015.22.252.6501

**Published:** 2015-11-18

**Authors:** Ephrem Tefera, Tariku Belay, Seleshi Kebede Mekonnen, Ahmed Zeynudin, Tefera Belachew

**Affiliations:** 1 Department of Medical Laboratory Sciences, College of Health and Medical Sciences, Haramaya University, Dire Dawa, Ethiopia; 2 Department of Medical Laboratory Sciences and Pathology, College of Public Health and Medical Sciences, Jimma University, Jimma, Ethiopia; 3 Department of Population and Family Health, College of Public Health and Medical Sciences, Jimma University, Jimma, Ethiopia

**Keywords:** Therapeutic efficacy, brand, albendazole, soil transmitted

## Abstract

**Introduction:**

Different brands Albendazole are commercially available and the efficacious brand/s is/are required for effective control of STHs infection. Thus, this study is aimed at determining the therapeutic efficacy of different brands of albendazole against soil transmitted helminths among school children of Jimma town.

**Methods:**

A cross sectional survey for prevalence of geohelminths and a randomized trial for efficacy study of different brands of albendazole was conducted among students Mendera Elementary School from March 29 to April 29, 2010. Positive subjects were randomized into three treatment arms using lottery method. The collected stool samples were examined by the McMaster method. CRs were calculated using SPSS windows version 16 and ERRs were calculated using appropriate formula.

**Results:**

Of the 715 school children who had their stools examined, 326 were positive for STHs with a prevalence rate of 45.6%. The cure rates (CR) for A. lumbricoides, T. trichiura and Hookworm were 99.4, 59.9 and 93.7%, respectively. Similarly, the egg reduction rates (ERR) were 97, 99.9 and 99.9% respectively. A statistical significant mean STH egg count difference were observed between pre and post-intervention study (p <0.001). But no statistical significant curing effect difference were observed among the three brands used against the three STHs (p >0.05).

**Conclusion:**

All the three brands of Albendazole tested regardless of the brand type were therapeutically efficacious for Ascariasis, Trichuriasis and Hookworm infections irrespective of the infection status whether it was single or multiple.

## Introduction

The three major soil transmitted helminths (STHs), A. lumbricoides N. americanus/A. duodenale (the hookworms), T. trichiura are amongst the most prevalent parasites worldwide. It is estimated that there are more than one billion cases worldwide, of which 450 million have significant morbidity attributable to their infection, the majority of whom are children [1]. *Ascaris lumbricoides* and *T. trichiura* are the commonest nematode parasites of man in Ethiopia. The hookworms, which most commonly infect humans, are Ancylostoma duodenale and Necator americanus. But in Ethiopia, N. americanus is significantly more common [2]. Infection intensity is a key factor in understanding the morbidity of STH; although light infections are often asymptomatic, heavy infections cause an array of morbidities, including dietary deficiencies and delayed physical and cognitive development [3]. According to WHO guidelines, intensity of infection was classified as “light”, “moderate” or “heavy” on the basis of fecal egg count [4]. Environmental sanitation is one of the methods that help to control geohelminthiases. However, in developing countries, it is difficult to prevent infection with geo-helminths because improvements in environmental sanitation are not easily achievable. Therefore, treatment of infected individuals with effective and broad-spectrum anthelminthics can minimize problems that arise from intestinal helminthic infections [5, 6]. As a result the availability of safe, effective, broad-spectrum anthelminthics that can be administered in single doses has changed the approach to the control of intestinal helminthiases [7]. Hence, Albendazole has the broadest spectrum of activity of the benzimidazoles released to date and has been widely used in human clinical medicine as a safe anthelminthic with high activity against larval and adult stages of nematodes and cestodes [8]. Efficacy of albendazole is usually measured using qualitative and quantitative diagnostic tests for eggs or larvae in feces at an optimal time interval post-treatment, which is dependent upon the species of each parasite. Cure rate (CR) and the fecal egg reduction rate (ERR) are indicators that are commonly used to measure the reduction in prevalence and reduction in intensity of infection [9]. Although the efficacy of albendazole is high against *A. lumbricoides* and moderate against hookworm, cure rates are unsatisfactory for *T. trichiura* [10]. Several studies reported that Albendazole is effective against *A. lumbricoides* and hookworms at a single day dose of 400mg administration. However, it is less effective against *T. trichiura* using the same dosage administration as compared to against *A. lumbricoides* and hookworms with a slight difference in cure rates and egg reduction rates from studies to studies [11–26]. Many of the studies conducted so far focused on either evaluating the efficacy of a single brand albendazole versus different helminths or the efficacy of albendazole in comparison to other anthelminthic benzimidazoles such as mebendazole versus a given helminth(s). Thus, the aim of the present study was to assess the therapeutic efficacy of different brands of albendazole that are commercially available in the Ethiopian Drug market against geohelminths among school children of Jimma town.

## Methods

Jimma town is located 335Km Southwest of Addis Ababa (The capital of Ethiopia) with a total area of 102 km^2^, in Jimma Zone, one of the eleven zones in Oromia Regional State. According to the 2007, population and housing census the town has a total population of 120,600, of which 60,590 are males and 60,010 are females. Temperature ranges from 12-30°C with a mean daily temperature of 19°C and the average annual rainfall is 800-2500 mm^3^. The town has an altitude of 1720-2010 m above sea level. The latrine coverage of the town is 91%. Also the town has water coverage of 84% [27, 28]. A cross sectional parasitological survey was conducted to determine the prevalence of geohelminths and a randomized trial was employed for efficacy study of different brands of albendazole among Students of Mendera Elementary school found in Jimma town from March 29 to April 29, 2010. The sample size was determined using a formula (Z^2^ P(1-P)/d^2^) for estimating single population proportion. The proportion of STHs among the school children was assumed to be 58% obtained from a study conducted by Amare et al. in 2007 [29] and the complement of the proportion was 42%. By considering a 95% confidence level and a 5% expected margin of error the calculated sample size was 374. To minimize errors arising from the probable occurrence of non compliance, 10% of the sample size was added to the calculated sample size thereby making it to be 411. Since Mendera Elementary School was selected from 12 governmental elementary schools found in the town using cluster sampling technique, there was a design effect of 2 that made the actual sample size 822. Then the total sample size were allocated to different grades i.e. grade 1 to 8 of Mendera Elementary School proportional to size of each grade and the sampling frame was the students’ enrollment list. To ensure randomization of the study subjects, random number table was employed to select them. As a result, the study subjects were selected from the list at random using table of random number. But out of the 822 sampled students 715 were voluntary to be involved in the study thereby making the response rate to be 87%. Finally from the 715 study participants, 326 students who were positive for geohelminths, were randomly allocated into three treatment arms (3 different treatment groups for the 3 brands of albendazole) using lottery method. However 8 students did not appear during the treatment days; hence only 318 were treated. Lastly those students who were allocated to the three treatment groups were documented by name, sex, age and grade level for the recollection of stool sample to conduct the efficacy study. Again of these 318 treated students, 10 were absent during the re-examination days. Hence net 308 students were involved in the efficacy study. For treatment all the three Brands of Albendazole: OVIS (South Korea, Manufactured by DAE HWA PHARM.CO., LTD), ALBENZ 400 (India, Manufactured by Sterling Lab) and WORMIN A 200 (Ethiopia, Manufactured by CADILA PHARMACEUTICALS (ETHIOPIA) PLC) … were purchased from local pharmacies after cross checking the Batch number given by DACA with that of the wholesalers invoice found in the private Pharmacies, this is because wholesalers are under the control of DACA.

During data collection all the study participants were interviewed whether they have been treated or not with any anthelmintics in the past three months and out of the 726 students 11 responded that they received anthelmintic drug in the past three months, as a result these 11 students were excluded from the study. Then all those who were positive for soil transmitted helminth, they were randomized into three treatment arms using random numbers obtained from random number table to be treated with a single dose (400mg) of different brands (Albenz-400 (India, Manufactured by Sterling Lab), Ovis (South Korea, Manufactured by DAE HWA PHARM.CO.,LTD) and Wormin A 200 (Ethiopia, CADILA PHARMACEUTICALS (ETHIOPIA) PLC)) of albendazole, and then 14 days later stool samples were collected and re-examined both for presence of geohelminth and for egg count. This is because, for STH's the interval between treatment and re-sampling should be not less than 7 days, and no greater than 21 days as per recommended by the WHO [1]. The rationale for this is that, this much time was considered to be a time that: excludes new infection after treatment as is it as long as the minimum pre-patent period and; ensures that all live and dead eggs from worms present at the time of treatment have had time to be expelled. It is assumed as per the WHO recommendation; each treatment arm will consist of 50 and more than 50 participants who are positive for geohelminth eggs [1]. To ensure randomization, allocation of the study participants to 3 treatment arm were carried out randomly using random numbers obtained from table of random number. Following randomization, 318 students were showed up on the treatment days out of the 326 STH positive students (7 were absent during the treatment days and one student refused to take the drug). Hence 104, 108 and 106 students were treated with a single dose three brands of 400mg Albendazole i.e. OVIS, ALBENZ 400 and WORMIN A 200 respectively by experienced Nurse under the supervision of the principal investigator. The dependent variables are cure rate, egg reduction rate and prevalence rate whereas age, sex, multiple infections by STH, mono-infections by STH, brand origin and the presence of other intestinal nematodes together with STHs are the independent variables. Two trained data collectors collected all the necessary data by using structured questionnaire. After the students being given adequate instruction on proper stool sample collection, all of them were provided with a stool cup, applicator stick and soft tissue paper to bring at least 2gm fresh stool sample of their own, which was sufficient for the McMaster method to count the eggs. Finally each sample was labeled and transported to Jimma University (JU) Clinical Lab Method Laboratory within half an hour together with filled questionnaire for processing and examination. Then all of the 715 stool samples collected were examined by the McMaster method by two experienced Laboratory Technologists. Data collection and microscopic examinations were under regular supervision. Data were entered into a computer then cleaned and analyzed using SPSS windows version 16. The egg reduction rate and cure rate of each drug were calculated using SPSS and appropriate formula respectively. Geometric mean egg count was estimated as exp (1n (c + 1)/n), -1 where c will be the egg counts (epg) for a particular individual and n is the number of students. Changes in egg counts within students will be compared by calculating Di = ln (co + 1)-ln (c1 + 1) for each individual, where 1n is natural logarithm, co is the egg count before treatment, c1 is the egg count after treatment and Di is the difference for the i^th^ student. Differences among the three treatments were compared using T-test and the percentage of egg count reduction induced by he treatment was estimated at 100(1-exp(-D))%, where D was the mean difference for a particular treatment. Furthermore, one way ANOVA was used to compare the efficacy of one brand in comparison to the other. Ethical approval was obtained from Ethical Review Board of Jimma University. Before the study was started, parents or guardians of the study subjects were gathered in different times and they were clarified about the objective of the study and finally signed on the consent form. In addition, verbal ascent was obtained from each of the study participants before they gave stool sample. Results were kept confidential. No other tests were done on the collected stool samples other than the mentioned one.

## Results

**Figure 1:**
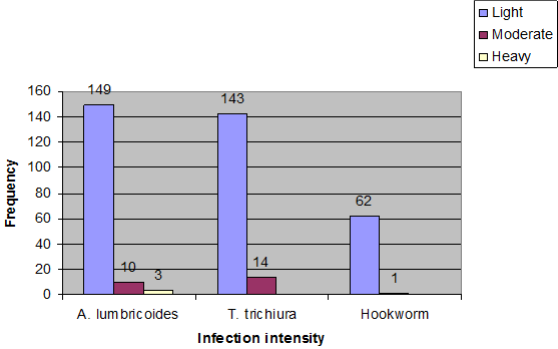
Figure showing intensity of soil transmitted helminths infection

Of the 715 school children who had their stools examined, 326 were positive for STHs with a prevalence rate of 45.6%. Out of the 715 study subjects 282 (39.4%) were males and 433 (60.6%) were females. Larger proportions (59.9%) of students were in the age range 10-14 years followed by those in the age group of 5-9 years (23.1%). Three hundred forty six students (48.4%) were found in grades 1 to 4 (first cycle) and the rest 369 (51.6%) were found in grades 5 to 8 (Second cycle). The most prevalent STH encountered was Ascaris lumbricoides 169 (23.6%) followed by Trichuris trichiura 165 (23.1%) and then by Hookworm 67 (9.4%). No heavy infections in the case of Trichuriasis and Hookworm were observed. The intensity of ascariasis, trichuriasis, and hookworm infections are indicated in figure (Figure 1). Curing effect of all the three brands of albendazole used did not vary by infection intensity of Ascariasis (p = 0.957). The same holds true for Hookworms (0.793). However there was a statistical significant curing effect difference among the brands used between light and moderate infection caused by *T. trichiura* (0.001) (Table 1). In this study the cure rates (CR) for A. lumbricoides, T. trichiura and Hookworm were 99.4%, 59.9% and 93.7%, respectively. Similarly, the egg reduction rates (ERR) were 97%, 99.9% and 99.9% for A. lumbricoides, T. trichiura and Hookworm, respectively (Table 2). There was a statistically significant difference in the soil transmitted helminths egg count between pre-intervention and post-intervention study (p < 0.001). This showed that all brands of Albendazole used were efficient enough either to reduce the egg production or to clear the parasites themselves (Table 3). Of the 318 geohelminth positive students, 104 were treated with OVIS (DAE HWA PHARM.CO., LTD), 108 were treated with ALBENZ 400 (Sterling Lab) and 106 were treated with WORMIN A 200 (CADILA PHARMACEUTICALS (ETHIOPIA) PLC). But 4 students from OVIS treated group, 2 from ALBENZ 400 treated group and 4 from WORMIN A 200 treated group were absent during the re-examination days. There is no a statistical significant curing effect difference among the three brands used against mono-infection caused by T. trichiura (p = 0.09). The same holds true for A. lumbricoides (p = 0.32) and for Hookworm (p = 0.82). This showed that there is no difference in the curing effect of all the three brands used against the three soil transmitted helminths (Table 4). Of the three brands of Albendazole, whose efficacy tested in this study, WORMIN A 200 is a locally produced generic product; whereas OVIS and ALBENZ 400 are imported brands from South Korea and India respectively. No statistical significant curing effect difference was observed between local and imported brands against Trichuriasis (p = 0.38). The same was true for A. lumbricoides (p = 0.13) and for Hookworm (p = 0.93) (Table 5). There was no statistical significant curing effect differences among the three brands used against mono-infection caused by T. trichiura (p = 0.10) and against double infection caused by T. trichiura and A. lumbricoides (Table 6). There was no statistical significant curing effect differences among the three brands used against mono-infection caused by Hookworm (p = 0.97) and against triple infection caused by all the three soil transmitted helminths (p = 0.66) (Table 7).

**Table 1:** Effect of three brands of albendazole in relation to infection intensity of STH among students attending Mendera Elementary School, Jimma, 2010

Infection Intensity	Students with ascariasis		Total	P-value
	not-cured	cured		
light infection	1(0.7%)	148(99.3%)	149(100.0%)	0.975
moderate infection	0(0.0%)	10(100.0%)	10(100.0%)	
heavy infection	0(0.0%)	3(100.0%)	3(100.0%)	
Total	1(.6%)	161(99.4%)	162(100.0%)	
**Infection Intensity**	**Students with trichuriasis**			
light infection	51(35.7%)	92(64.3%)	143(100.0%)	0.001
moderate infection	12(85.7%)	2(14.3%)	14(100.0%)	
Total	63(40.1%)	94(59.9%)	157(100.0%)	
**Infection Intensity**	**Students with hookworm infection**			
light infection	4(6.5%)	58(93.5%)	62(100.0%)	0.793
moderate infection	0(0.0%)	1(100.0%)	1(100.0%)	
Total	4(6.3%)	59(93.7%)	63(100.0%)	

**Table 2:** CR and ERR of different brands of albendazole against STH after 14 days of treatment among students attending Mendera Elementary School, Jimma, 2010

Parasitic species	Pretreatment	Post treatment 14 days			
	No of students positive for Ova	No of students cured	Cure Rate (%)	Geometric mean intensity (EPG)	Egg Reduction Rate %
*A. lumbricoides*	162	161	99.4	1.58	97
*T. trichiura*	157	94	59.9	63	99.9
Hookworm	63	59	93.7	5.05	99.9

**Table 3:** Distribution of egg counts before and after treatment among students attending Mendera Elementary School, Jimma, 2010

Parasitic Species	No of egg positive children	Mean(±SD)count before treatment	Mean(±SD)count After treatment	Mean difference	95%CI	P-value
*A. lumbricoides*	162	2051.85(±4387.55)	4.94(±62.85)	2046.91	1365.91-2727.92	<0.001
*T. trichiura*	157	436.24(±673.6)	139.49(±338.88)	296.82	228.44 - 365.19	<0.001
Hookworm	63	395.24(±393.26)	26.98(±165.79)	368.25	279.98 -456.53	<0.001

**Table 4:** Cure rates of three brands of albendazole against STH among students attending Mendera Elementary School, Jimma, 2010

Albendazole Brand used	Effect on *T. trichiura*		Total	P-value
	Not cured	Cured		
OVIS	18(29.5%)	43(70.5%)	61(100.0%)	
ALBENZ 400	22(48.9%)	23(51.1%)	45(100.0%)	0.09
WORMIN A 200	23(45.1%)	28(54.9%)	51(100.0%)	
Total	63(40.1)	94(59.9)	157(100%)	
Ascaris	**Effect on** ***A. lumbricoides***			
OVIS	0(0.0%)	48(100.0%)	48(100.0%)	
ALBENZ 400	0(0.0%)	64(100.0%)	64(100.0%)	0.32
WORMIN A 200	1(2.0%)	49(98.0%)	50(100.0%)	
Total	1(.6%)	161(99.4%)	162(100.0%)	
Hook worm	**Effect on Hookworms**			
OVIS	2(8.7%)	21(91.3%)	23(100.0%)	
ALBENZ 400	1(4.3%)	22(95.7%)	23(100.0%)	0.82
WORMIN A 200	1(5.9%)	16(94.1%)	17(100.0%)	
Total	4(6.3%)	59(93.7%)	63(100.0%	

**Table 5:** The cure rates of local brand versus imported brand against STH among students attending Mendera Elementary School, Jimma, 2010

Effect of local and imported drugs on parasitic clearance	Brand Type	Treatment effect		Total	P-Value
		Not cured	Cured		
*T. trichiura*	Imported	40(37.7%)	66(62.3%)	106(100.0%)	
	Local	23(45.1%)	28(54.9%)	51(100.0%)	0.38
	Total	63(40.1%)	94(59.9%)	157(100.0%)	
Hook worms	Imported	3(6.5%)	43(93.5%)	46(100.0%)	
	Local	1(5.9%)	16(94.1%)	17(100.0%)	0.93
	Total	4(6.3)	59(93.7)	63(100)	
*A. lumbricoides*	Imported	0 (0.0%)	112(100.0%)	112(100.0%)	
	Local	1(2.0%)	49(98.0%)	50(100.0%)	0.13
	Total	1(.6%)	161(99.4%)	162(100.0%)	

**Table 6:** Effect of three brands of albendazole against single and double infections by STH among students attending Mendera Elementary School, Jimma, 2010

Infection status	Brand type	Treatment outcome		Total	P-Value
Single infection		Not cured	Cured		
Students with Trichuriasis	OVIS	11(26.8%)	30(73.2%)	41(100.0%)	
	ALBENZ 400	15(51.7%)	14(48.3%)	29(100.0%)	0.10
	WORMIN A 200	17(41.5%)	24(58.5%)	41(100.0%)	
	Total	43(38.7%)	68(61.3%)	111(100.0%)	
**Double infection**		**Treatment outcome**			
Students both with Ascariasis and Trichuriasis	OVIS	7(35.0%)	13(65.0%)	20(100.0%)	0.43
	ALBENZ 400	7(43.8%)	9(56.2%)	16(100.0%)	
	WORMIN A 200	6(60.0%)	4(40.0%)	10(100.0%)	
	Total	20(43.5%)	26(56.5%)	46(100.0%)	

**Table 7:** Effect of three brands of albendazole against single and triple infections by STH among students attending Mendera Elementary School, Jimma, 2010

Infection Status	Brand Type	Treatment outcome		Total	P-Value
Single infection		Not cured	Cured		
Students with hookworm infection	OVIS	1(5.0%)	19(95.0%)	20(100.0%)	
	ALBENZ 400	1(4.5%)	21(95.5%)	22(100.0%)	
	WORMIN A 200	1(6.2%)	15(93.8%)	16(100.0%)	0.97
	Total	3(5.2%)	55(94.8%)	58(100.0%)	
**Triple infection**		**Treatment outcome**			
Students with all Ascariasis and Trichuriasis and hookworm infection	OVIS	1(33.3%)	2(66.7%)	3(100.0%)	
	ALBENZ 400	0(0.0%)	1(100.0%)	1(100.0%)	0.66
	WORMIN A 200	0(0.0%)	1(100.0%)	1(100.0%)	
	Total	1(20.0%)	4(80.0%)	5(100.0%)	

## Discussion

Comparative efficacy of different brands of albendazole was evaluated against ascariasis, trichuriasis and hookworm infections at 400mg single dose. The results showed that the CR and ERR of all the three brands for ascariasis were 99.4% and 97%, respectively. For trichuriasis it was 59.9% and 99.99% and for hookworm it was 93.7% and 99.9%, respectively. This study found a significant difference between pre and post intervention egg counts (mean count) (p = 0.001) thereby indicating that the drugs are most effective in either reducing the egg production or eliminating the parasite itself in the case of Ascariasis and Hookworm infections. But in the case of Trichuriasis the drugs are more or less effective because there was a statistical significant mean egg count difference between pre and post intervention counts (p = 0.001), though the CR (59.9%) was not comparable to that of Ascariasis and hookworm infection CRs (99.4% and 93.7% respectively) (Table 3). The CRs of OVIS, ALBENZ 400 and WORMIN A 200 for ascariasis were 100%, 100% and 98% respectively. For trichuriasis it was 70.5%, 51.1% and 54.9% respectively. Similarly for hookworm infection it was 91.3%, 95.7% and 94.1% respectively (Table 4). All the three brands of albendazole used were therapeutically efficacious irrespective of the worm burden harbored by the students in the case of Ascariasis and Hookworm infections. But they were more efficacious for light infection than moderate infection caused by T. trichiura (Table 1). The results indicated that Albendazole from the three brands (OVIS, ALBENZ 400 and WORMIN A 200) at 400mg single dose were effective against A. lumbricoides, T. trichiura and Hookworms and did not differ in cure rates (Table 4). But a similar study by Albonico et al. in 1994 reported that Zentel (SmithKline Beecham) was less effective against T. trichiura [11]. In our study, all the three brands of Albendazole given at a single dose of 400mg brought about low cure rate (59.9%) against Trichuriasis as compared to against Ascariasis (99.4%) and to against Hookworm infection (93.7%). However the egg reduction rates brought about by all the three brands of Albendazole against the soil transmitted nematodes were almost comparable, 97%, 99.99% and 99.9% against A. lumbricoides, T. trichiura and Hookworms respectively. In this study the CR of 99.4% obtained with a single dose 400mg in all the three brands together against Ascariasis was almost comparable with a cure rate over 97% (SmithKline Beecham (Zentel)) reported by Albonico et al. in 1994 [11]. The cure rate of 100% against ascariasis obtained with OVIS and ALBENZ 400 in the present study is consistent with a study done in Bolivia in 1993 with a cure rate of 100% [12]. But, the CR of 91.7% (Table 4) obtained with OVIS against hookworm is higher than a report from Bolivia (that of Bolivia is 81.8%), the most probable reason could be age variation of the students in Bolivia (2 to 9 years) when compared to the present study (5 to 19 years) [12]. The ERR of the present study 97%, 99.99% and 99.9% for Ascaris, Trichuris and hookworm respectively varied slightly with a study done in Thailand by Jongsuksuntigul et al. in 1993.(100%, 96% and 87%) [13]. The possible explanation for this is that, immune status variation of the study subjects of both studies. This is because the study participants in Thailand were adult patients. In our study a CR of 99.4% obtained against A. lumbricoides was comparable with a similar study in Tanzania by Hanspeter et al. in 1996 with a CR of 99%. But cure rates of 93.7% and 59.9% against Hookworm and T. trichiura observed in the present study were in disagreement with Hanspeter's report with cure rates 88.3% and 42.6% for hookworm and T. trichiura respectively. The reason could be due to difference in sample size; this is because our sample size 715 was very much larger when compared with Hanspeter's sample size 301. However, the egg reduction rates observed in our study 97%, 99.99% and 99.9% against A. lumbricoides, T. trichiura and Hookworm respectively were almost in agreement with Hanspeter's report with ERRs 99.7%, 92.2% and 98.5% [30]. In our study the prevalence of Hookworm was 9.4% among the school children; but it was reduced to 1.3% by day 14 after treatment. Previous report in Australia by Reynoldson et al. in 1998 indicated that the population prevalence of Hookworm fell from 80% to 1% by day 10-17 [15]. Both local and imported brands of albendazole were therapeutically efficacious for the three soil transmitted helminths, because no significant curing effect differences were found between local and imported brands against geohelminthic infections (Table 5). Though, there is little variation in cure rates when the drugs act against single versus multiple geohelminthic infections, still no statistical significant curing effect difference was found among the brands used (Table 6, 7). In all, the drugs were effective enough either in reducing the number of eggs to be produced by the parasites or to clear them off regardless of the brand origin whether it was imported brand or locally produced brand. The limitation of this study is that our inability to infer to the general population including all age groups; this is because our study involved school children as a study subject.

## Conclusion

We have shown that single dose 400mg albendazole is efficacious enough to treat ascariasis, hookworm infection and trichuriasis. All the three brands of Albendazole tested regardless of the brand type as well as the brand origin were therapeutically efficacious for Ascariasis, Trichuriasis and Hookworm infections irrespective of the infection status whether it was single or multiple. Also the three brands of Albendazole used had significant curing effect on students who had light infection intensity of Trichuriasis than students who had moderate infection intensity of Trichuriasis. So we suggest that all the brands irrespective of their origin can be used in a community based deworming program without considering the species of soil transmitted helminth involved as well as the polyparasitism observed in the infection. Moreover, further study also needed for more comprehensive data to be produced.
